# Evaluation of semen parameters from Fleckvieh–Simmental bulls and the influence of age and season of collection

**DOI:** 10.5194/aab-66-113-2023

**Published:** 2023-03-02

**Authors:** Radek Filipčík, Zuzana Rečková, Vojtěch Pešan, Oleksandra Konoval, Tomáš Kopec

**Affiliations:** 1 Department of Animal Breeding, Faculty of AgriSciences, Mendel University in Brno, Zemědělská 1, 613 00 Brno, Czech Republic; 2 Department of Animal products manufacturing and processing technology, Faculty of Livestock Raising and Water Bioresources, National University of Life and Environmental Sciences of Ukraine in Kyiv, Henerala Rodimtseva Str. 19, Building 1 of 12, 03401 Kyiv, Ukraine

## Abstract

The aim of this paper was to evaluate semen parameters from Czech Fleckvieh (dual-purpose breed) bulls used in artificial insemination in the Fleckvieh population. The ejaculate was collected from bulls only once a week, which is not usual. Respectively, the effects of age and season of collection on semen parameters were tested. The average volume of ejaculate by Fleckvieh bulls was 8.72 mL, which is higher than results in studies of bulls which were collected usually more than once a week. The average total motility was 72.82 % and progressive motility was 67.99 %. Sperm concentration reached on average $1254.10\times 10^{6}$ ${\mathrm{mL}}^{-1}$ . The total motility of spermatozoa after thawing was 40.88 %. A significant influence of age was observed on ejaculate volume, total and progressive motility, and total motility after thawing ( P<0.05 ). The season of collection had a significant influence on the volume of ejaculate, total motility, progressive motility, concentration of spermatozoa, and total count of spermatozoa ( P<0.01 ).

## 1 Introduction

Insemination is an effective tool for the dissemination of quality genetic material (Suyadi et al., 2020). Insemination can be considered one of the oldest biotechnological methods that have significantly affected animal production worldwide (Abeygunawardena et al., 2001). Currently, insemination is the most widely used reproductive technology with interesting benefits and costs (Oliveira et al., 2013). Artificial insemination is one of the most important reproductive biotechnologies, especially for dairy cattle, because the reproductive efficiency of dairy cows is very important for the economic success of dairy operations (Hagevoorth and Garcia, 2013). Even in beef production systems, good reproductive performance is essential for efficient management and production (Costa et al., 2011). The method also has disadvantages, namely that some bulls may excrete viruses in their semen without any clinical symptoms (Morrell, 2011). This reproductive technology allows animals to have more offspring, which reduces the need for parent animals (Jemal and Lemma, 2015).

There are many factors that can affect insemination. Bull fertility is one of the important factors in cattle reproduction (Hoflack et al., 2007). It is much more important than in cows because one bull can be used to breed cows in natural mating or potentially hundreds of thousands of times through artificial insemination (Kastelic, 2013). The property of sperm can affect the conception rate in dairy cows (Bilkis et al., 2016). The quality of sperm is important, which depends mainly on the choice of bull (Saacke, 2008). The quality and quantity of semen in bulls is responsible for a percentage of reproductive failure in cattle production (Tanga et al., 2021). Each bull has different quality sperm. When collecting sperm from certain bulls, the sperm may be of acceptable quality, but when cryopreserved, the sperm does not survive, as the freezing and thawing process can adversely affect the nucleus, plasma membrane, and acrosomal and mitochondrial membranes of sperm. It may adversely affect the processes required for successful fertilization (Makarevich et al., 2018). A Sperm Class Analyzer^®^ CASA system is used to evaluate the quality of fresh sperm, which is considered a better method due to faster measurement but also in terms of accurately measuring more dimensions of sperm fertility (Tanga et al., 2021). Sperm quality can be evaluated on its basic properties by macroscopic or microscopic examination (Agriris et al., 2018).

Bull semen quality parameters include volume, density, initial motility, sperm concentration, motility before filling, and motility after thawing (Sankhi et al., 2019). Some have added other parameters to this, namely the integrity of the sperm membrane and the integrity of the sperm chromatin. Poor sperm quality is associated with subfertility (Morrell, 2011). One of the most important criteria in quality assessment is sperm morphology (Vikazi and Webb, 2004). Sperm concentration is important to achieve optimal fertility during insemination. A low concentration of insemination doses leads to reduced viability after thawing. The reduction of sperm viability in doses may be affected by fresh sperm volume, sperm count, and seminal plasma levels after final dilution. The number of sperm needed to achieve optimal fertility is one of the main things for insemination (Mohanty et al., 2018). Sperm quality can also be affected by the age of bulls, as sperm quality in older bulls decreases with age due to testicular degeneration, which leads to loss of quality (Hoflack et al., 2007). The age of the bull can have a significant effect on volume, motility, concentration, and sperm production. As the age of the bulls increases, the volume of sperm increases up to the age of 7. On the contrary, from the age of 3 and with increasing age, the sperm concentration of the bull decreases (Agriris et al., 2018). The largest volume, motility, concentration sperm, and live sperm are usually obtained at the age of 5–7 years (Sankhi et al., 2019). The body enlarges and at the same time there is rapid growth of the testicles, so the amount of ejaculate increases in older animals. With increasing age, small sperm defects may appear, but in most cases the defects decrease with age (Stádník et al., 2014).

The season can also have a significant effect on cattle reproduction. Periods of high temperatures can lead to damaged spermatogenic cells which can result in testicular degeneration and reduced spermatogenesis efficiency, resulting in poor sperm quality (Hirwa et al., 2017). Sperm morphology is better in spring and winter than in autumn and summer (Vilakazi and Webb, 2004). In periods of drought such as spring and summer, it may result in a higher incidence of sperm head abnormalities, anomalies, and sperm counts, compared to the rainy season (Hirwa et al., 2017). Overall, it is known that sperm concentration, sperm count, and motile cells per ejaculate are higher in spring and winter than in summer. The seasonal effect is not easy to control, so it is important to know the other factors that are part of this effect, such as temperature, humidity, and day length, so that we have information for improved conditions in bulls (Mathevon et al., 1998).

The aim of this study was to describe the quantitative and qualitative semen parameters from Czech Fleckvieh bulls and evaluate the effect of age and season of collection on bull semen parameters.

## 2 Materials and methods

### 2.1 Sample collection

The quantitative and qualitative semen parameters from 46 Fleckvieh–Simmental bulls in artificial insemination (AI) station ISB Bohdalec (Czech Republic) were evaluated in our study. In total, 1029 samples were assessed using the Sperm Class Analyzer^®^ CASA system. All ejaculates were collected using the sampling method into an artificial vagina on a dummy. For sexual stimulation, bulls were allowed on false mounts and a standard artificial vagina was used (temperature of 42 ${}^{\circ }C$ ). Additional stimulation was provided by the collection team, consisting of a bull handler and a semen collector. All ejaculates were collected by the sampling team of the AI station. The analysis of semen was carried out immediately after its collection in the laboratory on the territory of the breeding station. The volume (mL) of semen, total motility (%), progressive motility (%), concentration ( $10^{6}$ ${\mathrm{mL}}^{-1}$ ), and total count of sperm ( $10^{6}$ ) were measured. After assessing the native ejaculate, it was diluted into individual insemination doses and frozen. The sample of doses of each bull was thawed after 24 h, and the total motility after thawing (%) was measured. The monitored period was from September 2019 to June 2021, and at least 10 samples per one bull were required. Thus, the lowest number of collections per bull was 10 and the highest was 57 samples; an average of 22 samples per one bull were obtained. In total, 1029 samples from 46 bulls were observed. The semen was collected once a week from bulls (varying from 6 to 8 d between each collection). Therefore, no effect of the collection interval was included in the model.

For the purpose of testing the effect of season of collection, the bulls were divided into four groups: winter, spring, summer, and autumn. There were 248 bulls in the winter group (December–February), 348 bulls in the spring group (March–May), 183 bulls in the summer group (June–August), and 250 bulls in the autumn group (September–November). The effect of age was tested by dividing bulls into four groups. The youngest group contained bulls up to 500 d old (278 bulls), in the second group there were bulls aged 501–750 d (304 bulls), in the third group there were bulls aged 751–1200 d (221 bulls), and the oldest group contained bulls above 1200 d old (226 bulls).

### 2.2 Statistical analysis

All descriptive statistics and general linear model (GLM) analysis were performed in SAS 9.1 program (SAS Institute, 2004). Pearson correlation coefficients and Box–Cox transformations were performed in the program Statistica 12 (TIBCO Software Inc, 2017). The volume and total motility after thawing showed a normal distribution. In the case of total motility, progressive motility, concentration, and total count of sperm, a Box–Cox transformation was performed before using GLM analysis for testing the significance of explanatory variables (Table 2).

The effect of the chosen factors (explanatory variables) were tested via the GLM formula as follows:

1 $$y_{ilkl}=\mu +{\text{age}}_{i}+{\text{ season}}_{j}+{\text{year}}_{k}+{\text{bull}}_{l}+e_{ijkl},$$

$y_{ilkl}$ is the dependent variable, μ is the intercept, ${\text{age}}_{i}$ is the i th effect of the age of the bull ( i=1 –4; 1: less than 500 d of age, 2: 501–750 d, 3: 751–1200 d, 4: above 1200 d), ${\text{season}}_{j}$ is the j th effect of the season of collection ( j=1 –4; 1: December–February, 2: March–May, 3: June–August, 4: September–November), ${\text{year}}_{k}$ is the k th effect of the year of collection ( k=1 –3, 2019–2021), ${\text{bull}}_{l}$ is the l th effect of the bull ( l=1 –46), and $e_{ijkl}$ is the random residual error.

## 3 Results

The descriptive statistics of the primary dataset are shown in Table 1. The general mean of the volume was 8.72 mL of ejaculate with a standard deviation of 3.15 mL, and the average total motility was 72.82 % (standard deviation of 14.11 %). The progressive motility was lower, with an average value of 67.99 % and a standard deviation of 14.94 %, which is close to the standard deviation of total motility. The average concentration of sperm per 1 mL was $1254.10\times 10^{6}$ ${\mathrm{mL}}^{-1}$ sperm, and the median was $1138\times 10^{6}$ ${\mathrm{mL}}^{-1}$ sperm. The total count of sperm in ejaculate had an average value of $10\;885.41\times 10^{6}$ , and the median value was $9879.67\times 10^{6}$ . The total motility after thawing was an average value of 40.88 %. The standard deviation was 12.14 % in the case of total motility after thawing. It can be concluded that the variability of total motility after thawing was lower than the total and progressive motility before freezing.

**Table 1:** Descriptive statistics of the dataset.

Variable	Median	Mean	SD	SEM	Min	Max
Volume (mL)	8.9	8.72	3.15	0.10	0.90	16.00
T. motility (%)	76	72.82	14.11	0.44	15.00	95.64
P. motility (%)	71	67.99	14.94	0.47	13.00	92.95
Concentration ( $10^{6}$ ${\mathrm{mL}}^{-1}$ )	1138	1254.10	643.92	20.12	63.05	4296.00
TCS ( $10^{6}$ )	9879.67	10 885.41	6729.93	210.83	160.56	43 113.60
T. motility a. t. (%)	40	40.88	12.14	0.41	5.00	81.00

SD: standard deviation, SEM: standard error of the mean, TCS: total count of sperm, T. motility a. t.: total motility after thawing, and P. motility: progressive motility

The effects of age, season, and year of collection and the effect of the bull were tested for each monitored parameter. The significance of these effects are shown in Table 2. All effects had a significant influence on volume, total motility, and progressive motility. The effect of the bull was the most important explanatory variable for all traits, the percentage of the explained total variability by the effect of the bull was from 29 %–30 % (for total motility after thawing) to 50 % (for total motility and progressive motility) and about 32 %–39 % in case of volume, concentration, and total count of sperm. It shows that for the total motility after thawing there was a lower impact of the bull's effect for these traits. The rest of the explanatory variables (season of collection, year of collection, and age of bulls) had a small impact on the total variability of the model (2 %–4 % of the total variability was explained) in all observed parameters.

**Table 2:** Significance ( p -values) of effects of explanatory variables on dependent variables.

Effect	Volume	T. motility	P. motility	Concentration	TCS	T. motility a. t.
Age	0.0209	0.0209	0.0339	0.1347	0.1001	0.0399
Season of collection	<0.0001	<0.0001	0.0001	<0.0001	<0.0001	0.3237
Year of collection	0.0117	0.0024	<0.0001	0.4554	0.2019	<0.0001
Bull	<0.0001	<0.0001	<0.0001	<0.0001	<0.0001	<0.0001
$R^{2}$	0.42	0.59	0.58	0.55	0.48	0.4

TCS: total count of sperm, $R^{2}$ : coefficient of determination of linear model, T. motility a. t.: total motility after thawing.

The effect of the age of the bulls at the collection time, divided into four age groups, had a statistically significant influence on the volume of ejaculate ( p=0.0209 ), total motility ( p=0.0209 ), progressive motility ( p=0.0339 ), and total motility after thawing (0.0399). In opposition, we could not reject a null hypothesis in case of the influence of age on the concentration and total count of sperm (respectively, p=0.1347 and p=0.1001 ). There was no significant effect of age on the concentration and total count of sperm. The effect of the season of collection (spring, summer, autumn, and winter) had a significant influence on volume ( p<0.0001 ), total motility ( p<0.0001 ), progressive motility ( p=0.0001 ), concentration ( p<0.0001 ), and total count of sperm ( p<0.0001 ). The season of collection had no statistically significant effect on the total motility after thawing.

The year of collection (2019–2021) had a statistically significant influence on volume ( p=0.0117 ), total motility ( p=0.0024 ), progressive motility ( p<0.0001 ), and total motility after thawing ( p<0.0001 ). No statistically significant effect of the year of collection was observed for concentration and total count of sperm (respectively, p=0.4554 and 0.2019). The differences among each animal were represented by the effect of the bull, this effect was statistically highly significant in all observed parameters ( p<0.0001 ).

### 3.1 The effect of the age of the bulls

Means and standard errors of the means for each level of the age factor are shown in Table 3. The first group includes bulls up to 500 d of age, the second group includes bulls 501–750 d old, the third group includes bulls 751–1200 d old, and the last group include bulls above 1200 d old. The volume, total motility, and progressive motility show continuous increases in the mean values in the first three age classes; then, the highest age group was decreasing in values. The means of volume increased from 7.52 to 9.73 mL from the first to third group of age, and in the fourth group decreased to 9.30 mL. The total motility increased from 69.26 % in the youngest bulls to 76.78 % in the third group, and in oldest group of bulls only 71.62 % was monitored, which is lower than in the second age group. The same trend could be observed in the case of progressive motility, where increases from 64.95 % to 71.75 % in the first three groups were estimated. In the oldest group, the progressive motility decreased to 66.15 %, which is close to the value in the youngest group of bulls.

**Table 3:** Means and standard error of the means of dependent variables according to different age classes.

Effect	Level	Volume		T. motility		P. motility		Concentration		TCS		T. motility a. t.	
		Mean	SEM	Mean	SEM	Mean	SEM	Mean	SEM	Mean	SEM	Mean	SEM
Age	<500 d	7.52	0.18	69.26	0.86	64.95	0.89	995.58	29.04	7632.80	294.09	39.98	0.80
	501–750 d	8.65	0.18	74.09	0.87	69.33	0.93	1172.26	32.54	9908.58	316.82	43.91	0.66
	751–1200 d	9.73	0.19	76.78	0.78	71.75	0.87	1264.65	38.91	11 963.92	374.53	42.53	0.94
	>1200 d	9.30	0.21	71.62	0.90	66.15	0.95	1666.15	50.90	15 100.95	574.44	35.48	0.86

TCS: total count of sperm, SEM, standard error of the mean, T. motility a. t.: total motility after thawing.

The concentration of sperm and total count of sperm showed continuous increasing of mean values in all age categories, from the youngest to oldest bulls. Specifically, the concentration increased from $995.58\times 10^{6}$ ${\mathrm{mL}}^{-1}$ in the youngest group to $1666.15\times 10^{6}$ ${\mathrm{mL}}^{-1}$ in the last age group. The same situation was observed in the case of the total count of sperm, where the increase was from $7632.80\times 10^{6}$ of sperm in the first group to $15\;100.95\times 10^{6}$ sperm in the last age group. The total motility after thawing increased from the first to the second age group (from 39.98 % to 43.91 %), then decreased to 42.53 % in the third group, and then to 35.48 % in the oldest group of bulls.

It could be stated that the values of all observed parameters increase with the age of the bulls until the age group of 751–1200 d, and then it decreases. The best parameters, except total motility after thawing, are reached at this age category, specifically at the age of bulls around 2.7–3 years.

### 3.2 The effect of the season of collection

The results of the evaluated parameters according to the different season of collection are shown in Table 4. Season is expressed as winter (December–February), spring (March–May), summer (June–August), and autumn (September–November). The volume of ejaculate was highest in autumn (8.89 mL) and the lowest in wintertime (7.87 mL). Respectively, the total motility and progressive motility have the same tendency; the highest was in summer (76.02 % and 71.92 %) and the lowest in autumn (71.51 % and 66.10 %). The concentration of sperm per milliliter reached values from $1139.49\times 10^{6}$ ${\mathrm{mL}}^{-1}$ in autumn, then increased to $1247.36\times 10^{6}$ ${\mathrm{mL}}^{-1}$ in winter, $1276.00\times 10^{6}$ ${\mathrm{mL}}^{-1}$ in spring, and the highest was in summer, $1379.74\times 10^{6}$ ${\mathrm{mL}}^{-1}$ . In the case of the total count of sperm, the count of sperm growth increased from winter ( $9889\times 10^{6}$ ) to $10\;170.56\times 10^{6}$ in autumn and $11\;307.40\times 10^{6}$ in spring, to the highest value $12\;454.54\times 10^{6}$ in summer. The total motility after thawing has the lowest average in autumn, 40.35 %. It then increases from 40.66 % in winter to a maximum of 42.24 % in summer.

**Table 4:** Means and standard error of the means of dependent variables according to different season classes.

Effect	Level	Volume		T. motility		P. motility		Concentration		TCS		T. motility a. t.	
		Mean	SEM	Mean	SEM	Mean	SEM	Mean	SEM	Mean	SEM	Mean	SEM
Season	Dec–Feb	7.87	0.19	72.37	0.80	67.06	0.87	1247.36	42.30	9889.56	409.64	40.66	0.91
	Mar–May	8.82	0.17	72.41	0.75	67.96	0.77	1276.00	34.63	11 307.40	376.70	40.80	0.72
	Jun–Aug	9.46	0.22	76.02	1.03	71.92	1.07	1379.74	49.54	12 454.54	478.96	42.24	0.92
	Sep–Nov	8.89	0.20	71.51	0.98	66.10	1.07	1139.49	37.18	10 170.35	421.10	40.35	0.79

TCS: total count of sperm, SEM: standard error of the mean, T. motility a. t.: total motility after thawing.

In general, in winter, the lowest values in volume of ejaculate and total count of sperm was reached. In autumn, there were lowest values in the case of total motility, progressive motility, concentration of sperm per milliliter, and total motility after thawing. No lowest values were observed in springtime. Also, no maximum values were observed in winter and spring. The highest values were in the summertime for total motility, progressive motility, concentration of sperm, and total motility after thawing. In autumn, the highest value in the volume of ejaculate was observed. Most of the parameters had highest values in the summer season, and highest values were mostly observed in the autumn season. In our study, the effect of motility after thawing showed no significance among seasonal categories, and on the other hand the season had significant influence on the volume of ejaculate.

### 3.3 Relationship among sperm parameters

The relationships among sperm parameters are expressed in Table 5, using Pearson correlation coefficients. Correlation between progressive and total motility was 0.99, there was a strong relationship between these two traits. A high correlation was between the total count of sperm and concentration (0.71). The total count of sperm had a moderate correlation to volume (0.54). A moderate correlation was also between total motility after thawing and, respectively, the total motility and progressive motility (0.48 and 0.49). All other parameters are uncorrelated between each other, the correlation coefficients reached values from - 0.15 to 0.19.

**Table 5:** Pearson correlation coefficients among dependent variables with p -values.

	P. Motility	Volume	Concentration	TCS	T. motility a. t.
T. motility	0.9888	0.0549	0.1897	0.1810	0.4860
	p=0.00	p=0.110	p=0.000	p=0.000	p=0.00
P. motility		0.0586	0.1307	0.1364	0.4949
		p=0.088	p=0.000	p=0.000	p=0.00
Volume			- 0.1228	0.5428	0.1103
			p=0.000	p=0.00	p=0.001
Concentration				0.7074	- 0.1532
				p=0.00	p=0.000
TCS					- 0.0667
					p=0.052

TCS: total count of sperm, SEM: standard error of the mean, T. motility a. t.: total motility after thawing.

## 4 Discussion

Suyadi et al. (2020) described the volume parameters in different seasons and age of bulls in Indonesia. They stated that the volume was constant from 2–4 years old, then slightly increased from 5–8 years old and again increased until 10 years old. A similar pattern was observed for sperm motility. On the other hand, sperm concentration was constant for all age categories. In our study, all these parameters of volume, sperm concentration, and motility continuously increased until the age category of 751–1200 d old (until 3 years), and above 1200 d old it slightly decreased (volume and motility) or increased (sperm concentration). A significant effect of season was also mentioned by Suyadi et al. (2020), but due to a different climate in Indonesia, there was a different trend.

Fuerst-Waltl et al. (2006) analyzed the effects of age and environmental factors on semen parameters of Austrian Simmental bulls. The volume of ejaculate was from 5.1 to 6.9 mL. These values are smaller than our results (8.72 mL) due to higher frequency of semen collection from the Austrian bulls. Semen samples analyzed in our research were collected once a week. The total number of spermatozoa in the Austrian population was from $4.0\times 10^{9}$ to $7.1\times 10^{9}$ , it was also less than in our study ( $9.9\times 10^{9}$ ). The effect of the class of bulls was statistically high, significant for volume, total count of spermatozoa, motility score, and concentration. These results are different compared to our study, where concentration and TCS were not significantly influenced by age. Zamuna et al. (2016) stated the volume of ejaculate by Simmental bulls was an average of 6.9 mL, these results are also lower compared to our study.

Zamuna et al. (2016) evaluated sperm parameters in different breeds, including the Simmental breed in Indonesia. The volume of ejaculate by Simmental bulls was 6.9 mL. The average of total sperm was $9421.3\times 10^{6}$ , which is similar to our results. Suchocki and Szyda (2015) analyzed sperm parameters in the Polish Holstein breed. The average volume was 4.18 mL, the concentration of sperm was $1471.21\times 10^{6}$ ${\mathrm{mL}}^{-1}$ , the total count of sperm was $6125.83\times 10^{6}$ , and the total motility was 72.40 %. Janicki and Cygan-Szczegielniak (2008) compared the bull parameters in three different AI stations. The volume ranged from 5.07 to 7.53 mL, the concentration of spermatozoa was from $966.37\times 10^{6}$ to $1626.40\times 10^{6}$ . The total motility before freezing ranged between 69.67 % and 76.67 %, and the total motility was between 50.00 % and 54.00 %.

Sankhi et al. (2019) described the effect of age on semen parameters. In this study, the bulls were divided to three age categories: 3–4, 5–7, and 8–9 years old. The volume of ejaculate was lowest in the first group at 5.12 mL, the second group had the highest volume of 6.72 mL, and the third group had 5.98 mL. This trend was similar to our results, but it is not comparable because of different age categories. In our research, younger bulls were included. The oldest age group from our study equals to the youngest group in the study by Sankhi et al. (2019). In this study, the highest concentration of sperm and motility were highest in the second age group. Our results also pointed to an increasing value of motility in the higher age group and decreasing of motility in the oldest age group. The concentration of sperm continuously increased from the youngest to oldest age category in our study. An analysis of bull semen parameters of different breeds in Ireland showed that the volume and concentration grew considerably from 10 months of age to 50 months of age (Berry et al., 2019).

Murphy et al. (2018) observed total motility before freezing and after thawing in different age categories in Holstein cattle. Both motilities were lowest in the category up to 1 year of age of the bulls. In other categories, there were similar values of total motility. The difference between youngest and other age categories was approximately 2 %. In the case of total motility before freezing, it showed the same trend compared to our results, but after thawing, the total motility was lowest in the last age category in our study. The lowest value of total motility in different ages was more than 80 % before freezing and more than 52 % after thawing in the study of Murphy et al. (2018). On the contrary, in our study, values of total motility in different ages were between 69.26 % and 76.78 % before freezing and from 39.98 % to 43.91 % after thawing. The volume of ejaculate showed a similar trend in a study by Murphy et al. (2018), when there was an increasing of volume of ejaculate from the youngest to oldest age category, but the values of volume were in general lower than in our study. It could be due to a higher frequency of sperm collection in the study by Murphy et al. (2018). For sperm concentration, there were different results in the study by Murphy et al. (2018), where up to 2 years of age the concentration increased and then slightly decreased.

Murphy et al. (2018) assessed the effect of the season on bull semen parameters. This study showed a significant effect on sperm concentration, total sperm count, and post-thaw motility. No significant influence of the season on the volume of ejaculate was observed.

Berry et al. (2019) described a phenotypic correlation among semen parameters from bulls in Ireland. The correlation between sperm concentration and volume of ejaculate was - 0.01. Between the volume and total count of sperm was a correlation of 0.63, and 0.68 was between the total count of sperm and the concentration. These results are comparable to our research.

## 5 Conclusion

Sperm parameters of Czech Fleckvieh bulls were comparable to other populations of dairy and dual-purpose breeds. Due to a long interval between semen collection (once a week), particularly the volume of ejaculate was higher compared to similar studies. The total motility of spermatozoa was 72.82 %, and 24 h after thawing, it decreased to 40.88 %. There was an observed statistically significant influence of environmental factors of the season of collection and also the influence of age and individuality of the bull on semen parameters. The effect of age had no statistically significant influence on spermatozoa concentration, and the seasonal effect did not show a significant influence on total motility after thawing.

## Data Availability

Data are available from the corresponding author upon request.
